# Somalian women with female genital mutilation had increased risk of female sexual dysfunction: a cross-sectional observational study

**DOI:** 10.1038/s41598-022-19949-0

**Published:** 2022-09-17

**Authors:** Abdikarim Hussein Mohamed, Rahma Yusuf Haji Mohamud, Hussein Ali Mohamud, Aşır Eraslan, Metin Gur, Abdikarim Ali Omar, Sertac Cimen

**Affiliations:** Mogadishu Somalia Turkish Training and Research Hospital, Mogadishu, Somalia

**Keywords:** Public health, Urology

## Abstract

Studies regarding the impact of female genital mutilation/cutting (FGM/C) on sexual function are scarce. This study is the first to explore the rate of female sexual dysfunction (FSD) among Somalian women who underwent FGM and its association with different FGM types. This study was carried out among women with a history of FGM who visited our clinic for a medical check-up. It relied on data including socio-demographic features, type of FGM determined by an examination, and the Female Sexual Function Index (FSFI) scores. Overall, 255 women were included. While 43.9% (n = 112) of the respondents had a history of Type 3 FGM, 32.2% had Type 2 (n = 82), and 23.9% had Type 1 (n = 61) FGM. Among all patients, 223 had FSD (87.6%)*.* There was a significant association between the FGM type and FSD (p < 0.001). The mean total FSFI score for the patients with Type 1, 2, and 3 FGM was 22.5, 19.7, and 17.3, respectively, all indicating FSD. The FSD is prevalent among mutilated Somalian women. Patients with Type 3 FGM had the lowest mean total FSFI scores indicating that the impact on sexual function was correlated with the extent of tissue damage during FGM.

## Introduction

Somalia has the highest rate of female genital mutilation (FGM) in the world (98%), followed by Guinea (96%), Djibouti (93%), and Egypt (91%)^1^. This practice is also called cutting, excision, or female circumcision. It is a destructive procedure performed for non-medical reasons that involve partial or total removal of the external female genitalia^2^. World Health Organization (WHO) classified FGM into four different types ranging from clitoridectomy (Type 1, Sunna), excision (Type 2), infibulation (Type 3, pharaonic), and Type 4 (piercing, pricking, scraping, incising, and cauterizing the genital area)^3^.

It has been widely practiced in several African and Asian countries for cultural, traditional, hygienic, and religious beliefs affecting about 125 million girls under fifteen^4^. It was reported that women who underwent FGM had psychosocial disabilities, sexual embarrassment, and low quality of life, impacting their psychological and physical well-being^5^. Thus, in addition to violating a fundamental human right, FGM also leads to anxiety, depression, and social isolation^3,4^. Furthermore, it is also associated with short-term and long-term complications, including bleeding, infection, dermoid cysts and abscesses, chronic pelvic pain, recurrent urinary tract infections, difficulties in menstruation, maternal and fetal issues, and dyspareunia^6^. It was also noted that the severity of these complications correlated with the type of FGM^6^. Fite et al. reported that women with FGM had lower sexual desire, less sexual satisfaction, and a higher risk of dyspareunia than other women^3^. In a systematic review of fifteen studies, including a total of 6672 women, Pérez-Lopeza et al. reported that FGM was associated with an increased risk of female sexual dysfunction (FSD)^2^.

Despite Somalia being the country with the highest prevalence of FGM worldwide, there have been no studies reported from Somalia regarding the rate of FSD among women who underwent FGM. Therefore, this is the first study to explore the rate of FSD among Somalian women who underwent FGM and the association of FSD with different FGM types.

## Method

This study was approved by the institutional ethical review board of Somalia Turkish Training and Research Hospital (approval number MSTH/7123). All participants gave oral and written informed consent for participation in the research.

The study was carried out among women with a history of FGM who visited our clinic for a check-up between 30 November 2020 and 30 November 2021. Sexually active women aged between 18 and 50 with a history of FGM were included. Post-menopausal women, pregnant women, and those who refused to participate in the study were excluded.

The research design was a cross-sectional observational interview-based study. The interviews were run by a gynecologist (R.Y.H.M.) who completed a standardized structured questionnaire (i.e., FSFI) and performed a physical examination to determine the type of FGM based on the World Health Organization (WHO) criteria.

Data including socio-demographic features such as age, marital status, education level, occupation, age at the time of FGM, type of FGM, and FSFI scores were collected.

The FSFI is a validated tool of a patient-reported outcome measure for assessing female sexual function^7,8^. It consists of 19 questions for six domains, including desire (2 questions), arousal (4 questions), lubrication (4 questions), orgasm (3 questions), satisfaction (3 questions), and pain (3 questions)^7^. The scores are determined based on patient responses covering the last 4 weeks. The total score ranges between 2 and 36, and FSD is considered in patients with a total score lower than 26.0^7,8^. All methods were carried out in accordance with relevant guidelines and regulations.

Statistical analyses were performed using the Statistical Package for Social Sciences (SPSS for Windows version 26, SPSS v26**, **IBM Inc., Armonk, NY, US) program. The data were analyzed by univariate descriptive statistics. The frequencies and percentages and the means ± standard deviations (SDs) were presented. Pearson's chi-square (χ2) test was applied to analyze the association between socio-demographic characteristics and the type of FGM. Shapiro–Wilk's test results showed that our scores were normally distributed, presented as means** ± **standard deviations (SDs), and compared using the one-way ANOVA and pairwise comparison for statistical analysis. The p value was considered significant when less than 0.05.

## Results

A total of 255 women were included. Most of the respondents (n = 112, 43.9%) had Type 3 FGM, while 82 (32.2%) had Type 2, and 61 (23.9%) had Type 1. There were no women with Type 4 FGM. The mean age of the participants was 24.3 ± 6.1 [18–50]. Most patients were aged between 18 and 30 (n = 167, 65.5%) or 31 and 40 (n = 69, 27.2%). Most participants (82.7%) were married. While 116 (45.5%) of the participants were employed, 113 (44.3%) were homemakers. Analysis of the age at which FGM was performed revealed that 138 (54.1%) of the respondents underwent this procedure at an age less than 8 years, while 109 (42.7%) were aged between 8 and 12.

Patient data, including age group, marital status, education level, occupation, and age at the time of FGM, and the association of these data with the types of FGM, are displayed in Table 1. There was a statistically significant association between the type of FGM and age group (χ^2^ p < 0.001), the level of education (χ^2^ p < 0.001), and age at the time of FGM (χ^2^ p = 0.041). These analyses revealed that women with a history of a more severe FGM were older than 30, illiterate, and had undergone the procedure at an age less than eight.

**Table 1:** Correlation of the socio-demographic characteristics with different types of FGM.

Variable	Type 1	Type 2	Type 3	p value
**Age**				< 0.001
18–30	48	66	53	
31–40	12	15	48	
41–50	1	1	11	
**Marital status**				0.104
Married	51	69	91	
Divorced	8	10	10	
Widow	1	2	11	
**Education**				< 0.001
Illiterate	17	27	60	
Primary school	16	9	21	
Secondary school	8	20	15	
University	20	26	16	
**Education**				0.189
Employed	35	39	42	
Homemaker	19	34	60	
Unemployed	6	8	10	
**Age during FGM**				0.041
< 8	24	45	69	
8–12	34	36	39	
> 12	3	1	2	

Type 1, Sunna: clitoridectomy, Type 2: excision, Type 3, pharaonic: infibulation.

FGM: female genital mutilation.

Statistical analysis was performed using Pearson's chi-square (χ^2^) test.

Assessment of the FSFI scores showed that more than two-thirds of the respondents had FSD (87.6%). There was a statistically significant association between the type of FGM and FSD in all domains and the mean total score. The severity of FSD was associated with the type of FGM. The mean total FSFI scores were 22.5, 19.7, and 17.3 for the FGM Type 1, 2, and 3, respectively. Since all these scores are lower than 26.0, they all indicate FSD. The association between the three types of FGM and the mean FSFI scores is shown in Table 2. The most affected domains in all three types were sexual orgasm and sexual satisfaction*.* The patients with Type 3 FGM had the lowest scores in all domains and the lowest total score. Our analysis revealed that FSD was present in 88.1% of the Type 3 FGM cases, 74.8% of the Type 2 FGM patients, and 56.2% of Type 1 FGM cases.

**Table 2:** Association of the FGMtypes with the mean FSFI scores.

Variable	Type 1	Type 2	Type 3	p value
Desire	3.2 ± 0.34	2.9 ± 0.56	2.2 ± 0.85	< 0.001
Arousal	4.8 ± 0.55	3.7 ± 0.47	3.1 ± 0.53	< 0.001
Lubrication	4.6 ± 0.45	3.5 ± 0.79	2.9 ± 1.43	< 0.001
Orgasm	3.4 ± 0.73	2.7 ± 1.16	2.1 ± 0.78	< 0.001
Satisfaction	3.7 ± 0.36	3.3 ± 0.95	2.8 ± 1.23	< 0.001
Pain	2.8 ± 0.65	3.6 ± 1.32	4.2 ± 0.84	< 0.001
Overall score	22.5 ± 2.51	19.7 ± 4.62	17.3 ± 5.24	< 0.001

The total score ranges between 2 and 36, and sexual dysfunction is considered in patients with a total score lower than 26.0

FGM: female genital mutilation; FSFI: Female Sexual Function Index.

### Institutional review board statement

The ethics approval form was received from the ethics committee of Mogadishu Somali Turkey Training and Research Hospital (approval number MSTH/7123).

### Informed consent statement

All patients obtained informed consent.

## Discussion

Somalia has the highest rate of FGM in the world, with 98% of the girls undergoing this counter-human rights procedure^1^. However, no studies have been reported from Somalia regarding the rate of FSD among women who experienced FGM. The current study showed that 87.6% of the patients with FGM had FSD. In a case–control study from Egypt, Ismail et al. worked on 197 women who underwent FGM^9^. They noted that 83.8% of these patients had FSD. This rate is close to the rate we found in our study.

Our study found that the mean total FSFI scores were 22.5, 19.7, and 17.3 for patients with Type 1, 2, and 3 FGM, respectively, indicating FSD in all types of FGM. In another case–control study from Egypt, 272 women with FGM were compared with the non-mutilated controls^10^. This comparative analysis revealed that the total FSFI score was significantly lower in patients with FGM (14.3 ± 5.9) than the healthy controls (25.9 ± 3.44)^10^. In addition, another study from Kenya reported that women with a history of FGM had lower FSFI scores, specifically in lubrication, orgasm, and satisfaction domains, compared to those without this history^11^. However, there was no difference between the two groups regarding sexual desire, arousal, and pain.

There are controversies in the studies regarding the sexual consequences of FGM^12–14^. However, Shafaati Laleh et al. reported in a comparative study regarding sexual function in 550 women from Iran that FGM significantly impacted lubrication and sexual satisfaction^12^. Also, these authors noted that discomfort and pain during sexual intercourse were more common in women with FGM than the others. However, the two groups were similar concerning arousal, desire, and orgasm.

Sexual pain during vaginal sexual intercourse is a common sequela of FGM^13,14^. Women with a history of FGM were reported to have a 1.5-fold increased risk of dyspareunia. It was also stated that Type 3 FGM was associated with the highest risk of sexual pain during vaginal sexual intercourse due to the challenges in fitting the penis through the small infibulated vaginal opening. Of note, clitoral neuromas or vulvar cysts may create vulvar and sexual pain in patients with a history of Type 1 or 2 FGM^13,14^. Our study revealed that the impact on sexual pain was correlated with the extent of tissue damage during FGM.

In our study, orgasm and satisfaction were the most affected domains in all types of FGM. Abdelhafeez MA et al. worked on 500 genitally mutilated Egyptian women and reported that these women had significantly lower sexual satisfaction and orgasmic function than those who were not mutilated^15^. In contrast, Zakaria Obaid et al. reported that patients who were genitally mutilated and non-mutilated differed only in terms of lubrication^16^.

The practice of FGM affects more than 200 million women aged between 15 and 49 worldwide^17^. Our study found that Type 1 FGM was more common among young patients, while Type 2 and 3 FGM were more prevalent among older women. A similar trend was detected in a study from Iran^18^*.* Again, in our study, most Type 3 FGM cases were elderly patients who were illiterate, unemployed, and underwent FGM before 8 years of age, indicating that this practice was more common in the twentieth century. In line with these findings, a systematic review including 54 published articles concluded that a low literacy level, maternal history of FGM, or belonging to the Muslim religion were significantly associated with an increased risk of undergoing FGM^19^.

Women with a history of FGM had dyspareunia, sexual embarrassment, vaginal dryness during sexual intercourse, orgasmic dysfunction, and dissatisfaction^20,21^. In addition, FGM is associated with a lack of sexual desire, low self-confidence, and self-esteem.

This study has some limitations that must be considered while evaluating its findings. First, it has no control group since most Somalian women undergo FGM. Second, this analysis did not include patients' comorbidities, the potential effect of socio-demographic differences among FGM groups on the results, and obstetric histories (including postpartum status) that can potentially impact their sexual function. Of note, if we had a control group, our data would be more reliable and reflect the impact of other variables, including sociocultural features, on sexual function. Also, since we did not include detailed obstetric histories and comorbidity status of the patients in our analysis, their impact on sexual function might be inadvertently attributed to FGM and have led to bias.

On the other hand, this study is the first to explore the rate of FSD among Somalian women with a history of FGM and the impact of different FGM types on their sexual life.

## Conclusion

The FSD is prevalent among genitally mutilated Somalian women, and its severity is related to the type of FGM. Patients with different FGM types had significantly different mean FSFI scores. Of note, patients with Type 3 FGM had the lowest mean FSFI scores indicating that the impact on sexual function was correlated with the extent of tissue damage during FGM. Since this practice has detrimental effects on female sexual function and women's psychosocial well-being and quality of life, it should be prevented by raising public awareness.

## Data Availability

The datasets used and/or analyzed during the current study are available from the corresponding author upon reasonable request.

## References

[1] Farouki, L. *et al.* The prevalence of female genital mutilation: A systematic review and meta-analysis of national, regional, facility and school-based studies. *medRxiv* (2022). 10.1371/journal.pmed.1004061 PMC9436112 36048881

[2] Pérez-López FR, Ornat L, López-Baena MT, Pérez-Roncero GR, Tajada-Duaso MC, Chedrau P (2020). Association of female genital mutilation and female sexual dysfunction: A systematic review and meta-analysis. Eur. J. Obstet. Gynecol. Reprod. Biol..

[3] Fite RO, Hanfore LK, Lake EA, Obsa MS (2020). Prevalence of female genital mutilation among women in Ethiopia: A systematic review and meta-analysis. Heliyon..

[4] Nnanatu CC, Atilola G, Komba P, Mavatikua L, Moore Z, Matanda D, Obianwu O, Kandala NB (2021). Evaluating changes in the prevalence of female genital mutilation/cutting among 0–14 years old girls in Nigeria using data from multiple surveys: A novel Bayesian hierarchical spatio-temporal model. PLoS ONE.

[5] Alinia C, Piroozi B, Jahanbin F, Safari H, Mohamadi-Bolbanabad A, Kazemi-Karyani A (2020). Estimating utility value for female genital mutilation. BMC Public Health.

[6] Horowicz M, Cottler-Casanova S, Abdulcadir J (2022). Diagnoses and procedures of inpatients with female genital mutilation/cutting in Swiss University Hospitals: A cross-sectional study. Reprod. Health.

[7] Hosseini SE, Ilkhani M, Rohani C, Nasrabadi AN, Gheshlagh RG, Moini A (2022). Prevalence of sexual dysfunction in women with cancer: A systematic review and meta-analysis. Int. J. Reprod. Biomed..

[8] Rincón-Hernández AI, Parra-Carrillo WC, Álvarez-Muelas A, Peñuela-Trujillo C, Rosero F, Espitia de la Hoz F, González JM, Saffon P, Vélez D, Franco E, Rojas M (2021). Temporal stability and clinical validation of the Spanish version of the female sexual function inventory (FSFI). Women Health..

[9] Ismail SA, Abbas AM, Habib D, Morsy H, Saleh MA, Bahloul M (2017). Effect of female genital mutilation/cutting; Types I and II on sexual function: Case-controlled study. Reprod Health..

[10] Mahmoud MIH (2016). Effect of female genital mutilation on female sexual function, Alexandria, Egypt. Alexandria J. Med..

[11] Esho T, Kimani S, Nyamongo I, Kimani V, Muniu S, Kigondu C (2017). The “heat” goes away: Sexual disorders of married women with female genital mutilation/cutting in Kenya. Reprod. Health..

[12] ShafaatiLaleh S, Maleki A, Samiei V, Roshanaei G, Soltani F (2022). The comparison of sexual function in women with or without experience of female genital circumcision: A case-control study in a Kurdish region of Iran. Health Care Women Int..

[13] Connor JJ, Brady SS, Chaisson N, Mohamed FS, Robinson B (2021). Understanding women’s responses to sexual pain after female genital cutting: An integrative psychological pain response model. Arch. Sex. Behav..

[14] Van de Velde SM, Van Eekert N (2021). Seeking a deeper understanding of the underlying causes of sexual pain in women who have undergone female genital cutting. Arch. Sex. Behav..

[15] Abdelhafeez M, Salem M, Eisa M (2020). Assessment of sexual troubles in Egyptian women with female genital mutilation. Evid. Based Women’s Health J..

[16] Obaid ZM, Amer AW-A, El Mahdy MAF, Mohammed AEB (2019). Evaluation of psychological and sexual effects of female genital mutilation (circumcision). Egypt J. Hosp. Med..

[17] Ahmed W, Mochache V, Stein K, Ndavi P, Esho T, Balde MD, Soumah AM, Diriye A, Ahmed MA, Petzold M, Pallitto C (2021). A hybrid, effectiveness-implementation research study protocol targeting antenatal care providers to provide female genital mutilation prevention and care services in Guinea, Kenya and Somalia. BMC Health Serv. Res..

[18] Daneshkhah F, Allahverdipour H, Jahangiry L, Andreeva T (2017). Sexual function, mental well-being and quality of life among kurdish circumcised women in Iran. Iran J. Public Health..

[19] El-Dirani, Z. *et al.* Factors associated with female genital mutilation: A systematic review and synthesis of national, regional and community-based studies. *BMJ Sex. Reprod. Health.* **48**(3), 169–178. 10.1136/bmjsrh-2021-201399 (2022). 10.1136/bmjsrh-2021-201399 PMC9279756 35264420

[20] Abdulcadir J, Catania L (2021). Conceptualizing sexual pain in women with female genital mutilation/cutting. Arch. Sex. Behav..

[21] Wilson AM, Zaki AA (2022). Novel clitoral reconstruction and coverage with sensate labial flaps: Potential remedy for female genital mutilation. Aesthetic Surg. J..

